# London Measure of Unplanned Pregnancy: guidance for its use as an outcome measure

**DOI:** 10.2147/PROM.S122420

**Published:** 2017-04-06

**Authors:** Jennifer A Hall, Geraldine Barrett, Andrew Copas, Judith Stephenson

**Affiliations:** 1 Research Department of Reproductive Health, UCL Institute for Women’s Health; 2 Department of Infection & Population Health, UCL Institute of Epidemiology and Health Care, London, UK

**Keywords:** ordinal outcomes, multivariate regression, London Measure of Unplanned Pregnancy, pregnancy intention, pregnancy planning, epidemiology

## Abstract

**Background:**

The London Measure of Unplanned Pregnancy (LMUP) is a psychometrically validated measure of the degree of intention of a current or recent pregnancy. The LMUP is increasingly being used worldwide, and can be used to evaluate family planning or preconception care programs. However, beyond recommending the use of the full LMUP scale, there is no published guidance on how to use the LMUP as an outcome measure. Ordinal logistic regression has been recommended informally, but studies published to date have all used binary logistic regression and dichotomized the scale at different cut points. There is thus a need for evidence-based guidance to provide a standardized methodology for multivariate analysis and to enable comparison of results. This paper makes recommendations for the regression method for analysis of the LMUP as an outcome measure.

**Materials and methods:**

Data collected from 4,244 pregnant women in Malawi were used to compare five regression methods: linear, logistic with two cut points, and ordinal logistic with either the full or grouped LMUP score. The recommendations were then tested on the original UK LMUP data.

**Results:**

There were small but no important differences in the findings across the regression models. Logistic regression resulted in the largest loss of information, and assumptions were violated for the linear and ordinal logistic regression. Consequently, robust standard errors were used for linear regression and a partial proportional odds ordinal logistic regression model attempted. The latter could only be fitted for grouped LMUP score.

**Conclusion:**

We recommend the linear regression model with robust standard errors to make full use of the LMUP score when analyzed as an outcome measure. Ordinal logistic regression could be considered, but a partial proportional odds model with grouped LMUP score may be required. Logistic regression is the least-favored option, due to the loss of information. For logistic regression, the cut point for un/planned pregnancy should be between nine and ten. These recommendations will standardize the analysis of LMUP data and enhance comparability of results across studies.

## Background

In 2012, 85 million women experienced an unintended pregnancy: 40% of all pregnancies globally.^1^ This was in part a consequence of the fact that 222 million women worldwide are not using an effective method of contraception, despite not wanting a child in the near future.^2^ Fully meeting the need for family planning could reduce maternal deaths by a further 30%,^3^ neonatal deaths by 0.6 million per year, and later infant deaths by 0.5 million per year, predominantly in low-income countries.^2^

Reducing unintended pregnancy and its adverse effects on maternal and neonatal outcomes remains a high priority for global reproductive health. In order to meet the need for family planning globally fully, we must develop a better understanding of women’s pregnancy intentions and behaviors. Improving contraceptive use is the mainstay of these efforts, as effective family planning programs should lead to a reduction in unplanned pregnancies. Similarly, effective preconception care should lead to an increase in planned pregnancies. However, currently there are challenges in the measurement of pregnancy intention as an outcome measure.

Most current estimates of levels of unintended pregnancy are derived from questions used in population-based surveys, such as the National Survey of Family Growth and the Pregnancy Risk Assessment Monitoring System in the US and Demographic and Health Surveys in low-income countries. For example, Demographic and Health Surveys ask a single question of women up to 5 years after their last birth to determine whether that pregnancy was intended or unintended.

However, pregnancy intention has increasingly been recognized as a complex concept that encompasses “affective, cognitive, cultural and contextual dimensions”.^4^ The aforementioned methodologies are unsatisfactory, as they diminish a complex concept to two categories, are likely to introduce recall bias, and overestimate intention, because reported intention may be greater after delivery then during pregnancy^5^ and abortions are omitted. While these surveys have provided useful information over the last 100 years, there has been increasing discussion of the limitations of these methodologies and of the need to develop a more sophisticated way of measuring the complex construct that is pregnancy intention.^4^,^6^–^12^

**Figure 1:**
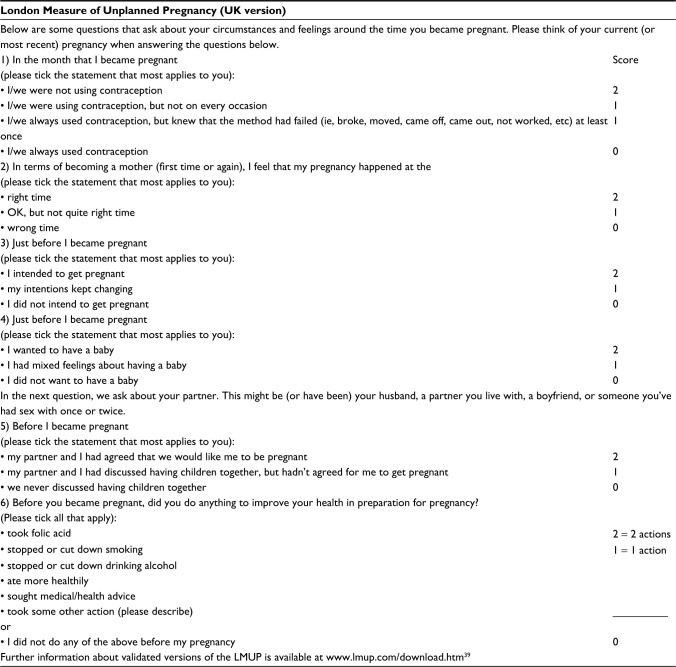
LMUP questions and scoring. **Note:** Reproduced from Barrett G, Smith S, Wellings K. Conceptualisation, development and evaluation of a measure of unplanned pregnancy. *J Epidemiol Community Health*. 2004;58(5):426–433.^7^ **Abbreviation:** LMUP, London Measure of Unplanned Pregnancy.

The London Measure of Unplanned Pregnancy (LMUP) is a psychometrically validated measure of the degree of intention/planning of a current or recent pregnancy.^7^ The LMUP is officially a measure of both pregnancy planning and intention, making no distinction between these broad concepts, consistent with the qualitative evidence underpinning the development of the measure. We also use the terms “planning” and “intention” interchangeably in this paper. As shown in Figure 1, LMUP comprises six questions, each scored 0, 1, or 2. These are summed to create an ordinal variable on a scale of 0–12, with each increase in score reflecting an increase in pregnancy intention. At first publication, provisional guidance about the interpretation of the scores was given by Barrett et al to aid the production of prevalence estimates (0–3, unplanned; 4–9, ambivalent; 10–12, planned); however, they recommended using the full scale in analysis.^7^

The LMUP has been formally and informally validated in multiple and diverse settings,^13^–^17^ and is increasingly being used as a research tool.^18^–^25^ There are multiple potential uses for the LMUP, but when used to evaluate the effectiveness of family planning or preconception care programs it is a patient reported outcome measure (PROM). A recent consensus statement has recommended the LMUP as an outcome measure for preconception care in the US.^26^

**Figure 2:**
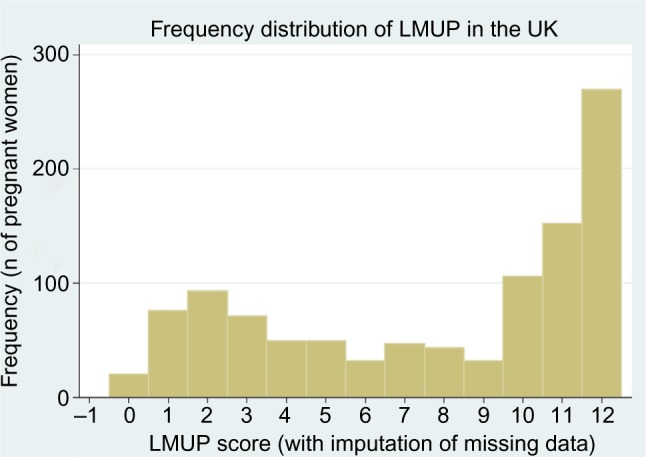
The distribution of LMUP scores in the original UK data. **Abbreviation:** LMUP, London Measure of Unplanned Pregnancy.

**Figure 3:**
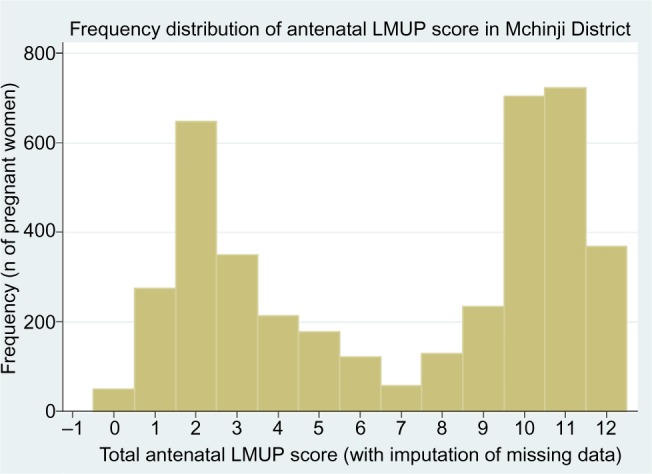
The distribution of LMUP scores in our Malawi data. **Note:** Reproduced from Hall JA, Barrett G, Phiri T, Copas A, Malata A, Stephenson J. Prevalence and determinants of unintended pregnancy in Mchinji District, Malawi; using a conceptual hierarchy to inform analysis. *PLoS One*. 2016;11(10):e0165621.^32^ **Abbreviation:** LMUP, London Measure of Unplanned Pregnancy.

Pregnancy intention is a strongly socially patterned phenomenon, and the distribution of LMUP scores is affected by the composition of the sample. In the original UK validation study,^7^ with a sample closely matching the UK population of pregnant women, the distribution of LMUP scores was negatively skewed, probably bimodal (Figure 2). This distribution has been replicated in other large, population-based UK studies.^21^,^22^ In other studies around the world, the distribution has consistently been abnormal,^14^,^15^,^17^ and bimodality is seen in our current Malawi data (Figure 3).

Beyond recommendations for the use of the full LMUP scale,^7^ there is no published guidance on how to use the LMUP as an outcome measure in analyses. Ordinal logistic regression has been recommended on the LMUP website,^27^ essentially the LMUP handbook, on the basis of unpublished PhD analyses (unpublished data, Barrett, 2002). Four studies using the LMUP to explore determinants of pregnancy intention to date have all used binary logistic regression, but have dichotomized the scale at different cut points.^22^–^24^,^28^ There is thus a need for evidence-based guidance to provide a standardized methodology for multivariate analysis using the LMUP as a PROM and to enable comparison of results.

There are several reasons why the choice of regression method for the multivariate analysis of the LMUP as an outcome measure is not immediately apparent. These include the non-Normal distribution of pregnancy intention and the ordinal nature of the LMUP score. In addition to the recommended ordinal logistic regression with the full score range and the binary logistic regression used in publications to date, linear regression and ordinal logistic regression with LMUP scores grouped into categories are also possibilities.^7^ Each model has its own advantages and disadvantages.

The linear model has the advantages of relative simplicity, use of the full range of LMUP scores, and ease of interpretation. However, using a linear model assumes that the relationship we are looking at is linear and that each interval on the scale is equivalent. This may not be the case for the LMUP, ie, the difference between pregnancies that score 3 and 4 may or may not be the same as the difference between pregnancies that score 10 and 11. In addition, for the model to be valid, the residuals should be Normally distributed and independent and the variance of the residuals should be constant. Treating the ordinal score as linear may violate the assumption that the variance of the LMUP score is homogeneous across the variables of interest, and while the parameter estimate may be unbiased, the estimates of variance may be biased and inconsistent.^29^

Binary logistic models require conversion of the LMUP score from an ordinal to a binary outcome. Until recently, this was the most common approach in situations where the outcome is ordinal categorical. However, there are two main limitations to this approach. First, it results in a loss of information, as categories are collapsed^30^ – and in the case of the LMUP, collapsing 13 categories to two results in the loss of a lot of information – and thus typically a loss of power to investigate relationships.^30^ Second, the choice of cutoff is not always obvious, and the results can be sensitive to the choice.^31^ Simulations have shown that the optimal cut point in terms of efficiency is considered to be where the cut creates two groups with equal numbers, ie, at the median, and that this model is asymptotically 75% efficient compared to an ordinal regression of a five-point scale^31^ (it will be less efficient for a 13-point scale like the LMUP). However, this is an arbitrary cut point, making the results difficult to interpret and of little practical use. An alternative would be to choose a cut point that is hypothesized to be relevant on the basis of theory, eg, a cut point at 9 for the LMUP, above which pregnancies would be described as “planned”.^7^ Introducing a cut point in this way is arbitrary, and the high starting number of categories in the LMUP score exacerbates the arbitrariness for the LMUP, suggesting that ordinal regression might be preferable.^31^

Ordinal logistic regression is a newer technique that has increasingly been used since the commands became available in common statistical packages. It was developed in recognition of the aforementioned limitations of collapsing ordinal scores to binary outcomes and of the growing amount of health data that were being collected on ordinal scales, eg, of pain or quality of life.^29^ There are two main types of ordinal regression: the proportional odds model and the continuation ratio model. The proportional odds model is the model most commonly used, is available as standard in Stata, and is provisionally recommended for use in the multivariate analysis of the LMUP when it is used as the dependent variable (unpublished data, Barrett, 2002).

The theory behind the proportional odds model (also called the cumulative odds model) is an extension of the logistic model for binary data, and is based on the assumption that there is an underlying continuous variable from which the ordered categorical variable is created.^31^ The proportional odds model calculates cut point-specific odds ratios at each cut point, using all observations in the data every time, but at a different level of dichotomization. As such, a five-point ordinal scale would have four cut points: comparing the first category to the last four categories, the first two categories to the last three categories, the first three categories to the last two categories, and finally the first four categories to the last category. From this, one summary odds ratio is calculated, based on the maximization of the likelihood function, which is valid over all cut points simultaneously.^29^ This means that inferences can be made across the range of outcomes considered, whereas the results of the binary logistic regression are confined to one cut point. This model is based on the assumption of homogeneity of odds ratios across each cut point.

Though ordinal logistic regression can be applied, retaining all values of the LMUP, which has been recommended and retains all possible information, these values may also be aggregated for analysis. This may be because problems are found or expected with convergence or precision when fitting ordinal logistic regression models with all values of the LMUP score, due to small cell counts. We have been unable to find any guidance on how many cut points can be managed by an ordinal logistic regression and the pros and cons of this choice in terms of “power” or “sensitivity” to detect associations. Another reason to aggregate is for simplicity and to link the regression analysis to meaningful prevalence estimates. With this in mind, we explore the application of ordinal logistic regression retaining all values of the LMUP and also with LMUP scores aggregated into three categories that seem theoretically valid: a score of 0–3 is classed as “unplanned”, 4–9 as “ambivalent”, and ≥10 as “planned”.^7^

The aim of this paper is to make recommendations for the analysis of future studies using the LMUP as an outcome variable. To do this, we used data from pregnant women in Malawi to compare different multivariate regression models for examining determinants of pregnancy intention with the LMUP score as the dependent variable. We also present a confirmatory analysis using LMUP data from a separate UK dataset: the original LMUP validation study.^7^ The recommendations would apply equally where the LMUP is used as an outcome measure in an interventional trial, eg, of a preconception care intervention.

## Materials and methods

The Malawi dataset comprised 4,244 pregnant women aged 15–49 years who were recruited in Mchinji District between March and December 2013. They were interviewed at their homes, and were two and nine months pregnant at the time of interview. The cohort has been described in more detail elsewhere.^32^ The UK dataset, which has also been described in detail elsewhere,^7^ comprised 1,039 women with a valid LMUP score, of whom 555 (53.4%) were currently pregnant and continuing their pregnancy to term, 221 (21.3%) were currently pregnant and opting for abortion, and 263 (25.3%) were postnatal. The UK dataset contained data on fewer of the potential determinants of pregnancy intention. The variables available were mother’s age, education level, birth order of the child, ethnicity, and whether she was living with her husband or partner. The stability of LMUP scores between pregnancy and the postnatal period was formally assessed and shown to be highly stable in the original UK psychometric analyses.^7^ As expected, women who were opting for abortion were mainly in the first trimester and tended to have low LMUP scores. Among women who were currently pregnant and continuing their pregnancies to term, there was no correlation between gestation and LMUP score (*r*=−0.03, *P*=0.32).

In total, five different multivariate-regression models were compared on the Malawi data: linear regression, binary logistic regression with a cut point at the median “Log med” or at an LMUP score of nine “Log plan”, and ordinal logistic regression using the full LMUP scale “LMUP all” or using the LMUP grouped into three categories (“LMUP 3”). First, the univariate relationship of each potential determinant of pregnancy intention with LMUP score was considered using each type of regression analysis and the results compared across the models. The potential determinants of pregnancy intention were developed on the basis of the literature, and are shown in Box 1. In the Malawi dataset, “tribe” consisted of the majority Chewa tribe, used as the baseline, and Senga, Ngoni, Yao, and “other”, whereas the UK data used “white British” as the baseline compared to “white other”, “black British”, Asian, and “mixed/other”. Religion was grouped with non-Catholic Christian as the baseline and Catholic Christian, Muslim, and “other” as the comparison groups. Intimate partner violence was measured using the Abuse Assessment Screen.^33^ Previous episodes of depression were assessed by asking women whether they had experienced low mood and/or anhedonia, and if so whether this lasted for more than 2 weeks.

Box 1 **Potential determinants of pregnancy intention**

**Table:** 

Socioeconomic status	Previous episodes of depression
Woman’s education (years)*	Intimate partner violence
Partner’s education (years)	Woman’s age (years)*
Partner’s age (years)	Number of live children*
Marital status*	Primiparity
Tribe/ethnicity*	Birth interval (years)
Religion	Gestation (months)
Distance to health facility (km)	

**Note:**

* Variables available in the UK dataset.

Multivariate models were created using each type of regression and including all variables. The coefficients (or odds ratios) of each variable were compared across the five models and classified the “same” if the coefficients were consistent in their direction and also either consistently statistically significant or consistently not significant across all the models. Otherwise, the coefficients were classified as “different”. This fairly crude distinction is for illustrative purposes only in the comparison of the different models; even when coefficients are “different”, the differences are often qualitatively very small.

The assumptions underlying each model were then formally tested. For the linear regression this involved checking that the standardized residuals are Normally distributed and that there is homoscedasticity of the variance of the residuals, and that there was proportionality of odds across response categories for the ordinal logistic regression models. The validity of the proportional odds assumption for both ordinal logistic regression models was also formally tested. In our regression models, following the standard approach, categorical covariates are represented by a set of binary indicator variables. If there are n categories, then there will be n−1 indicator variables, each representing the difference between a category and the category that is chosen to be the baseline. When testing the proportional odds assumption for a categorical variable, it is difficult to conduct a single test, and thus we tested the assumption for each indicator variable in turn. These tests assess whether the odds ratios for a category relative to the baseline are common across the cut points in the regression model. Where assumptions were found to have been violated, ways to address this were considered. For linear regression, this was the calculation of robust standard errors, and for the ordinal regression it was the calculation of a partial proportional odds model.

As a confirmatory analysis, the same methods were subsequently followed for the analysis of the UK LMUP data, though the Log med model was omitted, as this had been dropped during the analysis of the Malawi data. Whichever model is chosen, continuous covariates may be included as simple linear terms or more complex forms, such as spline functions. In this work, linear terms are used, and the linearity of association between LMUP score and each covariate was checked graphically. All analyses were conducted in Stata 13.

### Ethics approval and consent to participate

The UCL Research Ethics Committee and the College of Medicine Research Ethics Committee at the University of Malawi granted ethical approval for the research from which these data are drawn (3974/001 and P.03/12/1273, respectively). All participants gave written informed consent to take part in this research. Ethical approval for the original UK LMUP validation study was granted by a National Health Service multicenter research-ethics committee.

## Results

### Univariate analyses

A summary of univariate results is presented in Table 1. For most variables, there were no differences among types of regression, though there were some small variations in the size, but not direction, of the estimated effects. In general, the ordinal logistic models had the most precision (narrowest confidence intervals [CIs]), but the differences between the models were small and nonsignificant. For example, in the linear regression, each additional year of maternal education was associated with an increase in LMUP score of 0.15 (95% CI 0.11–0.18), indicating a more planned pregnancy. In the Log plan model, each additional year of maternal education increased the odds of a planned pregnancy by 1.06 (95% CI 1.04–1.09). The Log med model has a less intuitive interpretation, in that each additional year of maternal education increased the odds of having an LMUP score above the median by 1.07 (95% CI 1.04–1.09). In the ordinal regression models, the LMUP all model shows that each additional year of maternal education increased the odds of having an LMUP score above each point of the scale by 1.07 (95% CI 1.05–1.09). Finally, the LMUP 3 model tells us that the odds of having a planned pregnancy compared to an unplanned or ambivalent pregnancy, and also the odds of a planned or ambivalent pregnancy compared to an unplanned pregnancy, were 1.06 (95% CI 1.04–1.08) for each additional year of maternal education. These interpretations are the same for the multivariate models, except that the coefficients were then controlled for other variables in the model.

### Multivariate regression

The results of the five multivariate regressions are shown in Table 2. Values in bold were significant at *P*<0.05.

### Assessment of linear regression

**Figure 4:**
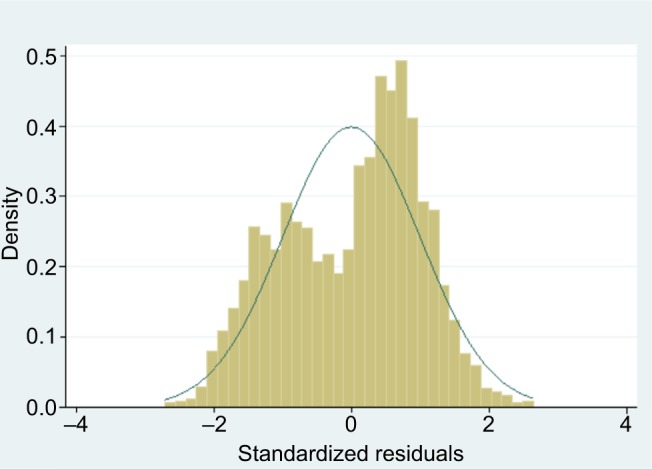
Standardized residuals from linear regression of our Malawi data.

**Figure 5:**
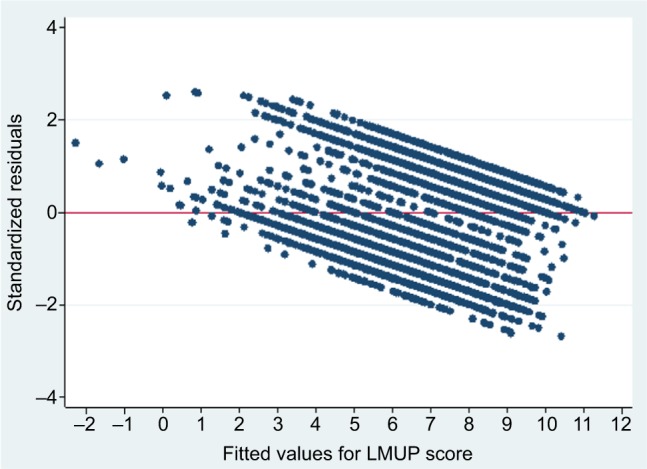
Scatterplot of standardized residuals against predicted values to show the variance of residuals from linear regression of our Malawi data. **Abbreviation:** LMUP, London Measure of Unplanned Pregnancy.

The standardized residuals were non-Normally distributed (Figure 4), the variance of residuals was not constant across the values predicted by the model, and the mean of the residuals was positive for low predicted values and negative for high predicted values (Figure 5), meaning that the assumptions were violated for the linear regression model. This is to be expected when applying linear regression to an outcome that is restricted to a range (here 0–12), and the problems are also evident in predicted values outside this range (Figure 5). We note, however, that predicted values outside the range occurred only at the lower end, leading to negative values, and also these occurred only rarely (five of 4,244). Investigation of these five negative values revealed that these women were unusual in their clustering of a number of extreme values across several variables in an atypical combination (unmarried, short birth interval, large number of children, and previous depression). These women would thus have been expected to have highly unplanned pregnancies.

Given a large sample size, it is possible to relax the assumptions slightly^34^ and, to help accommodate the non-Normal distribution of the residuals and the heteroscedasticity of the variance, robust (or Huber–White) standard errors can be calculated.^34^ The calculation of these standard errors makes no assumptions about the underlying probability model, but instead estimates them from the variability in the data. This method tends to result in larger standard errors and wider CIs. The result of these violations is that while the model is suitable to assess the existence of associations, there may be some slight errors in the estimations of the coefficients and their standard errors. We found broadly linear associations between continuous factors and the LMUP when plots were examined, and thus retained simple linear terms in our models.

### Assessment of logistic binary regression

Some differences were observed with regard to which factors were statistically significant between the two binary logistic models, underlining the impact of selecting the cut point and importance of selecting a cut point that is scientifically meaningful. However, the differences seen between odds ratios were generally modest. The cut point at the theoretically valid division of pregnancies into intended and unintended was more justifiable than the data-driven median cut point, and was taken forward for further consideration.

### Assessment of ordinal logistic regression

There were some minor differences with regard to which factors were statistically significant in each model, such as no tribe being significantly different from Chewa in LMUP all but the Ngoni tribe being significantly different to Chewa in LMUP 3. Tests confirmed that both models violated the proportional odds assumption at *P*<0.001.

### Development of partial proportional odds ordinal logistic regression model

Comparing models where all variables were constrained to the proportional odds assumption with models where no variables were constrained confirmed that the proportional odds assumption was invalid for at least one variable in both the LMUP all and LMUP 3 models. Therefore, partial proportional odds ordinal logistic regression, where the assumption of proportional odds is relaxed for some variables, was attempted for both the full LMUP scale and the LMUP in three groups. However, the LMUP all model could not be fitted without a large proportion of the women having a negative outcome probability, and was thus dropped.

### Selection of type of multivariate regression model

We identified three potential regression models to investigate further: linear regression using robust standard errors, binary logistic regression at the “planned” pregnancy cut point, and a partial proportional odds ordinal logistic regression model using the LMUP score grouped into three. The coefficients and odds ratios for these models are shown in Table 3. The variables for which the proportional odds assumption had to be relaxed, of which there were six, are shown in italics. These variables had different odds ratios across the two cut points. By relaxing the assumption of proportional odds, we are able to see which variables are associated with pregnancy intention in each of the categorizations and how their effect size differs across these cut points (shown in italics in Table 3), which is of interest in itself.

The findings are relatively consistent across the models, and for variables where the findings are labeled as different, these differences are generally modest. The partial proportional odds ordinal logistic regression model is the “best” model, as it is flexible and its assumptions have not been violated, but each model has different strengths and weaknesses.

### Analysis of UK LMUP dataset

Univariate analysis, shown in Table 4, found very minimal differences with regard to which variables were statistically significant across the models. The results of the four multivariate regressions – linear, Log plan logistic regression, and the two ordinal regressions LMUP all and LMUP 3 – are shown in Table 5. Those shown in bold were significant at *P*<0.05. The findings were the same for all variables in every model, with the exception of ethnicity, where there were a few small differences.

For linear regression, while the distribution of the residuals was roughly Normal, the variance and mean were not constant across the range of predicted values, as was also seen in the Malawi data (data not shown). There were no predicted values outside the range of 0–12. The only difference between the two ordinal logistic models was that being of Asian ethnicity was statistically significantly associated with LMUP score in the LMUP all model, but not in the LMUP 3 model. The LMUP all model violated the proportional odds assumption, whereas there was some evidence that the LMUP 3 model violated the assumption (*P*=0.075). Again, a partial proportional odds model could not be fitted for the full LMUP score. For the LMUP 3 model, relaxing the assumption of proportionality of odds for one indicator variable (not living with husband or partner relative to living with husband) resulted in a model that did not violate the assumption of proportional odds for any other covariates (data not shown).

## Discussion

While the assumptions of Normality of standardized residuals and constant variance were violated for the linear regression of the Malawian data, robust standard errors, which allows a model that contains heteroscedastic residuals to be fitted, can be used. We note also that predicted values outside the range 0–12 occurred rarely in this data and not at all in the UK data. The linear model has two significant advantages over the other models. First, it uses the full range of LMUP scores from 0 to 12, and second the results enable you to see how women vary across the LMUP scale. For example, using the linear regression on the Malawi data, we can say that on average an unmarried woman has an LMUP score that is 3.72 (95% CI 3.06–4.37) points lower than a married woman, having controlled for the other variables in the model (Table 2).

The main drawback of the binary logistic model, using nine as the cut point above which the pregnancy is considered “planned”, is the resultant loss of information and efficiency, having converted the ordinal 13-point scale to a binary outcome. It also only gives us an estimate of effect over one cut point.

It was not possible to calculate a stable partial proportional odds ordinal logistic regression model using the whole LMUP score in either dataset, meaning that the scores had to be collapsed to the three groups. This again resulted in a loss of information and efficiency; however, this gives estimates of effect across two cut points, as opposed to one, as in the binary logistic model. The interpretation of these odds ratios is arguably less intuitive. For example, in the Malawi data, for number of live children, which does not violate the proportional odds assumption and thus has the same odds ratios across both cut points, we can see that for every additional child, a woman in the unplanned or ambivalent group had 0.69 (95% CI 0.64–0.73) the odds of being in the ambivalent or planned group, respectively (Table 3). For mothers aged 15–17 relative to 18–29 years, a variable that does not have proportional odds, women had 0.45 (95% CI 0.33–0.61) the odds of being in the ambivalent or planned groups rather than in the unplanned group and 0.57 (95% CI 0.43–0.77) the odds of being in the planned group rather than the unplanned or ambivalent groups.

When the proportional odds assumption is violated, then this also raises some concerns over the validity of the linear regression. For example, our findings suggest that the effect of mother’s age is different when changing from unplanned to ambivalent compared to changing from ambivalent to planned. This calls into question the assumption in linear regression of a constant effect of mother’s age across all values of the LMUP.

There are few studies that have compared different types of regression or cut points on the same data. Norris et al compared linear, logistic, and ordinal regression models, using two different cut points for logistic regression and the proportional odds model, to analyze quality-of-life data.^35^ They found that linear and ordinal regressions had “similar and smaller confidence end-point ratios [the upper CI divided by the lower CI, a measure of parameter stability] when compared to the binary logistic models”, indicating that these models were more precise. It should be remembered, though, that these two models are not strictly comparable, as in the logistic regressions the size of the CI depends in part on the magnitude of the odds ratio. They also noted that the interpretation of these models was simpler. However, no one model is de facto better than any other, and the choice of model should depend on the aim of the analysis and considerations of model goodness of fit.

### Limitations

This paper has focused on statistical issues surrounding the use of the LMUP as an outcome measure. However, the models we considered do not allow a consideration of causality, which would require more sophisticated analyses. Nevertheless, the points raised here will be useful for researchers considering these analyses. Furthermore, this paper did not address the issues of using the LMUP as an independent variable, where similar difficulties with regard to the correct choice of analysis models may apply.

## Conclusion

Our analysis has shown that there are no important differences in findings between different regression models using LMUP score as the outcome variable. This was true for two separate datasets. We recommend that linear regression is used as a first-line analysis, even though the assumptions of constant mean and variance of the residuals across fitted values were violated in both datasets, because the full range of the LMUP score is used and for ease of analysis and interpretation. Researchers may have discounted this approach, given the nature of the LMUP score; however, the use of robust standard errors where needed can help to account for the violation of some of the assumptions behind a linear regression model.

Researchers could explore ordinal logistic regression using the full range of LMUP scores with their own data, but they may find that violation of the assumptions of this model requires a partial proportional odds ordinal logistic model to be fitted instead. This may further require the LMUP score to be collapsed to three groups, resulting in loss of information. Binary logistic regression is the least-favored option, given the loss of information. Where this option is chosen, we recommend using the standard cut point of 9/10 to distinguish between unplanned and planned pregnancies.

Unplanned pregnancies may be associated with a range of adverse outcomes for the mother and baby,^36^–^38^ and their reduction is a common aim of public health programs. The growing number of studies using the LMUP to measure pregnancy intention is a testament to its increasing recognition as a more valid outcome measure than those used to date. The use of the LMUP score allows us to develop a more nuanced understanding of women’s pregnancy intention and the determinants of unplanned pregnancy, meaning that prevention programs can be better tailored and targeted to women’s needs. The recommendations made in this paper support the expanding use of the LMUP by providing guidance for analyses using the LMUP to improve standardization and comparability of results. This will facilitate the use of the LMUP as a PROM to evaluate family planning and preconception care programs.

## Figures and Tables

**Table 1:** Findings from the univariate analyses of our Malawi data for the five different regression models

Variables	Type of regression model									
	Linear		Log med		Log plan		LMUP all		LMUP 3	
	β-Coefficient	95% CI	OR	95% CI	OR	95% CI	OR	95% CI	OR	95% CI
**Mother’s age, years**	18–29 as baseline									
15–17	−1.03	−1.46 to −0.59	0.63	0.50 to 0.78	0.79	0.64 to 0.99	0.66	0.54 to 0.80	0.61	0.50 to 0.76
≥30	−1.31	−1.59 to −1.03	0.54	0.47 to 0.62	0.52	0.45 to 0.61	0.57	0.50 to 0.64	0.56	0.49 to 0.64
**Father’s age, years**	20–29 as baseline									
15–19	−2.02	−2.69 to −1.35	0.38	0.27 to 0.55	0.46	0.32 to 0.66	0.43	0.32 to 0.58	0.38	0.27 to 0.53
≥30	−0.98	−1.23 to −0.73	0.62	0.54 to 0.70	0.58	0.51 to 0.66	0.65	0.58 to 0.72	0.64	0.57 to 0.71
**Mother’s education level, years**	0.15	0.11 to 0.18	1.07	1.04 to 1.09	1.06	1.04 to 1.09	1.07	1.05 to 1.09	1.06	1.04 to 1.08
**Father’s education level, years**	0.07	0.03 to 0.10	1.02	1.01 to 1.05	1.03	1.01 to 1.05	1.03	1.02 to 1.05	1.03	1.01 to 1.04
**Marital status**	Married as baseline									
Unmarried	−3.40	−3.89 to −2.97	0.16	0.12 to 0.21	0.19	0.14 to 0.26	0.24	0.20 to 0.29	0.18	0.15 to 0.23
**Number of live children**	−0.53	−0.60 to −0.47	0.77	0.74 to 0.80	0.74	0.72 to 0.77	0.79	0.76 to 0.81	0.78	0.75 to 0.80
**Primigravida** (yes)	1.43	1.17 to 1.69	1.92	1.68 to 2.20	2.59	2.26 to 2.97	2.02	1.79 to 2.27	2.16	1.9 to 2.46
No	Baseline									
**Intergestational period**	Baseline									
<2 years										
2–3 years	1.44	1.10 to 1.79	1.99	1.66 to 2.40	1.73	1.43 to 2.10	1.86	1.59 to 2.18	1.90	1.60 to 2.24
3–4 years	2.04	1.64 to 2.44	2.94	2.37 to 3.65	2.27	1.82 to 2.82	2.39	1.99 to 2.88	2.36	1.94 to 2.87
4–5 years	2.70	2.19 to 3.22	3.89	2.93 to 5.17	2.64	2.00 to 3.48	3.11	2.46 to 3.92	3.11	2.43 to 4.00
>5 years	2.55	2.04 to 3.06	3.71	2.81 to 4.90	2.84	2.16 to 3.72	2.92	2.31 to 3.68	3.10	2.41 to 3.99
**Socioeconomic status**	Poorest 20% as baseline									
Second-poorest 20%	0.35	−0.03 to 0.74	1.16	0.96 to 1.41	1.1	0.9 to 1.33	1.15	0.97 to 1.36	1.16	0.97 to 1.38
Middle 20%	0.47	0.09 to 0.86	1.24	1.02 to 1.50	1.11	0.91 to 1.35	1.20	1.02 to 1.42	1.25	1.04 to 1.49
Next-richest 20%	0.74	0.35 to 1.12	1.39	1.15 to 1.69	1.31	1.08 to 1.59	1.40	1.18 to 1.65	1.37	1.14 to 1.64
Richest 20%	0.83	0.45 to 1.21	1.38	1.14 to 1.67	1.30	1.07 to 1.57	1.52	1.28 to 1.81	1.39	1.16 to 1.66
**Previous depression**	None as baseline									
One/two for less than 2 weeks	−0.95	−1.28 to −0.61	0.58	0.49 to 0.69	0.61	0.51 to 0.72	0.69	0.60 to 0.81	0.64	0.55 to 0.75
One for more than 2 weeks	−1.93	−2.29 to −1.57	0.36	0.30 to 0.44	0.41	0.33 to 0.50	0.45	0.38 to 0.53	0.44	0.37 to 0.52
Both for more than 2 weeks	−2.23	−3.30 to −1.16	0.30	0.16 to 0.54	0.37	0.20 to 0.68	0.38	0.24 to 0.62	0.45	0.27 to 0.73
**Distance to health facility (km)**	No statistically significant differences in any model									
**Gestation (months)**	−0.10	−0.18 to −0.02	0.95	0.91 to 0.99	0.93	0.90 to 0.97	0.96	0.93 to 1.0	0.95	0.91 to 0.98
**Religion**	No statistically significant differences in any model									
**Tribe**	In all models, the Senga tribe was the only one statistically significantly different to baseline (Chewa)									

**Abbreviations:** LMUP, London Measure of Unplanned Pregnancy; CI, confidence interval.

**Table 2:** Comparison of the five multivariate regression models using our Malawi data

Variables	Linear		Log med		Log plan		LMUP all		LMUP 3		Model comparison
	β-Coefficient	95% CI	OR	95% CI	OR	95% CI	OR	95% CI	OR	95% CI	
**Mother’s age, years**	18–29 as baseline										
15–17	−**1.10**	−**1.56 to** −**0.63**	**0.54**	**0.40 to 0.74**	**0.54**	**0.39 to 0.74**	**0.60**	**0.47 to 0.76**	0.50	0.37 to 0.66	Same
≥30	0.39	0.00 to 0.78	**1.32**	**1.02 to 1.71**	**1.36**	**1.05 to 1.76**	**1.25**	**1.02 to 1.52**	1.24	0.99 to 1.54	Different
**Father’s age, years**	20–29 as baseline										
15–19	−**1.44**	−**2.10 to** −**0.78**	**0.45**	**0.29 to 0.69**	**0.43**	**0.28 to 0.69**	**0.47**	**0.34 to 0.65**	**0.40**	**0.27 to 0.59**	Same
≥30	**0.48**	**0.16 to 0.81**	1.22	0.98 to 1.51	**1.33**	**1.07 to 1.65**	**1.26**	**1.07 to 1.49**	**1.29**	**1.07 to 1.55**	Different
**Mother’s education level, years**	−0.01	−0.05 to 0.03	0.98	0.95 to 1.01	**0.96**	**0.94 to 0.99**	1.00	0.97 to 1.02	0.98	0.96 to 1.01	Different
**Father’s education level, years**	−0.03	−0.06 to 0.01	0.98	0.96 to 1.00	0.98	0.96 to 1.01	0.99	0.97 to 1.01	0.98	0.96 to 1.00	Same
**Unmarried**	−**3.71**	−**4.16 to** −**3.25**	**0.10**	**0.07 to 0.15**	**0.10**	**0.07 to 0.15**	**0.15**	**0.12 to 0.20**	**0.11**	**0.08 to 0.14**	Same
**Number of live children**	−**0.76**	−**0.87 to** −**0.64**	**0.61**	**0.57 to 0.66**	**0.62**	**0.57 to 0.68**	**0.70**	**0.66 to 0.74**	**0.68**	**0.64 to 0.73**	Same
**Birth interval**	First birth as baseline										
Within 24 months	−**2.07**	−**2.45 to** −**1.68**	**0.35**	**0.27 to 0.45**	**0.26**	**0.20 to 0.34**	**0.37**	**0.30 to 0.45**	**0.28**	**0.22 to 0.35**	Same
2–3 years	−**0.74**	−**1.14 to** −**0.34**	0.79	0.60 to 1.04	**0.49**	**0.38 to 0.65**	**0.66**	**0.54 to 0.81**	**0.52**	**0.41 to 0.66**	Different
More than 3 years	0.33	−0.08 to 0.74	**1.72**	**1.29 to 2.30**	0.87	0.66 to 1.15	1.05	0.86 to 1.29	0.88	0.68 to 1.12	Different
**Gestation, months**	−0.06	−0.13 to 0.01	0.97	0.92 to 1.02	0.97	0.92 to 1.02	0.99	0.95 to 1.02	0.97	0.93 to 1.01	Same
**Socioeconomic status**	Poorest 20% as baseline										
Second-poorest 20%	−0.13	−0.49 to 0.22	0.86	0.68 to 1.08	0.82	0.65 to 1.04	0.90	0.75 to 1.07	0.89	0.72 to 1.09	Same
Middle 20%	−0.13	−0.49 to 0.22	0.87	0.69 to 1.10	0.82	0.64 to 1.04	0.88	0.74 to 1.05	0.92	0.75 to 1.14	
Next-richest 20%	0.31	−0.04 to 0.67	1.07	0.84 to 1.36	1.03	0.81 to 1.31	1.05	0.88 to 1.26	1.09	0.89 to 1.34	
Richest 20%	0.33	−0.05 to 0.71	1.01	0.78 to 1.31	0.97	0.74 to 1.25	1.02	0.84 to 1.24	1.02	0.81 to 1.27	
**Previous depression**	None as baseline										
1 or ≤2 weeks	−**0.85**	−**1.17 to** −**0.54**	**0.55**	**0.44 to 0.67**	**0.52**	**0.42 to 0.64**	**0.62**	**0.53 to 0.73**	**0.59**	**0.49 to 0.71**	Same
1 or ≥2 weeks	−**1.34**	−**1.68 to** −**1.00**	**0.38**	**0.30 to 0.48**	**0.42**	**0.33 to 0.54**	**0.52**	**0.43 to 0.62**	**0.49**	**0.40 to 0.60**	
Both ≥2 weeks	−**1.65**	−**2.69 to** −**0.61**	**0.34**	**0.17 to 0.68**	**0.36**	**0.17 to 0.74**	**0.38**	**0.22 to 0.65**	**0.52**	**0.29 to 0.92**	
**Distance to health facility**	<2.5 km as baseline										
2.5–4.99 km	0.15	−0.24 to 0.54	**1.38**	**1.03 to 1.86**	**1.42**	**1.05 to 1.92**	1.22	0.97 to 1.53	1.23	0.95 to 1.60	Different
5–7.49 km	0.22	−0.15 to 0.59	**1.37**	**1.02 to 1.86**	**1.54**	**1.14 to 2.10**	1.17	0.93 to 1.48	1.26	0.97 to 1.64	Different
>7.5 km	0.18	−0.20 to 0.56	1.26	0.91 to 1.74	1.38	1.00 to 1.93	1.11	0.86 to 1.42	1.16	0.87 to 1.53	Same
**Religion**	Non-Catholic Christian as baseline										
Catholic	−0.11	−0.35 to 0.12	0.96	0.81 to 1.13	1.11	0.94 to 1.31	1.00	0.88 to 1.14	0.97	0.84 to 1.12	Same
Muslim	−0.05	−1.11 to 1.01	0.77	0.38 to 1.54	0.58	0.29 to 1.17	0.75	0.43 to 1.30	0.66	0.36 to 1.23	
Other	0.09	−0.81 to 0.99	1.02	0.53 to 1.93	0.79	0.41 to 1.49	0.92	0.59 to 1.45	0.89	0.53 to 1.51	
**Tribe**	Chewa as baseline										
Ngoni	−**0.56**	−**1.01 to** −**0.11**	**0.70**	**0.51 to 0.95**	**0.72**	**0.53 to 0.99**	0.86	0.68 to 1.09	**0.73**	**0.56 to 0.96**	
Senga	**1.16**	**0.64 to 1.68**	0.97	0.57 to 1.64	1.01	0.60 to 1.71	1.07	0.72 to 1.60	1.05	0.66 to 1.68	
Yao	0.17	−0.89 to 1.23	1.17	0.58 to 2.33	1.19	0.6 to 2.38	1.17	0.68 to 2.01	1.26	0.69 to 2.31	
Other	0.10	−0.80 to 1.00	1.12	0.61 to 2.07	0.94	0.52 to 1.69	0.91	0.57 to 1.45	0.94	0.56 to 1.59	Same

**Note:** Figures in bold denote significance (*P*<0.05).

**Abbreviations:** LMUP, London Measure of Unplanned Pregnancy; OR, odds ratio; CI, confidence interval.

**Table 3:** Comparison of three multivariate regression models using our Malawi data

Variables	Linear regression with robust standard errors		Binary logistic regression at “planned” cut point		Ordinal: unplanned to ambivalent and planned combined		Ordinal: unplanned and ambivalent combined to planned		Model comparison
	β-Coefficient	95% CI	OR	95% CI	OR	95% CI	OR	95% CI	
**Mother’s age, years**	18–29 as baseline								
15–17	−**1.10**	−**1.54 to** −**0.65**	**0.54**	**0.40 to 0.74**	***0.45***	***0.33* to *0.61***	***0.57***	***0.43* to *0.77***	Same
≥30	**0.39**	**0.02 to 0.76**	**1.32**	**1.02 to 1.71**	1.20	0.97 to 1.49	1.20	0.97 to 1.49	Different
**Father’s age, years**	20–29 as baseline								
15–19	−**1.44**	−**2.08 to 0.80**	**0.45**	**0.29 to 0.69**	**0.44**	**0.30 to 0.64**	**0.44**	**0.30 to 0.64**	Same
≥30	**0.48**	**0.24 to 0.72**	1.22	0.98 to 1.51	**1.32**	**1.10 to 1.58**	**1.32**	**1.10 to 1.58**	Different
**Mother’s education level, years**	−0.01	−0.05 to 0.03	0.98	0.95 to 1.01	0.99	0.97 to 1.01	0.99	0.97 to 1.01	Same
**Father’s education level, years**	−0.03	−0.06 to 0.00	0.98	0.96 to 1.00	0.98	0.96 to 1.00	0.98	0.96 to 1.00	Same
**Unmarried**	−**3.71**	−**4.37 to 3.05**	**0.1**	**0.07 to 0.15**	**0.13**	**0.1 to 0.18**	**0.13**	**0.1 to 0.18**	Same
**Number of live children**	−**0.76**	−**0.88 to 0.64**	**0.61**	**0.57 to 0.66**	**0.69**	**0.64 to 0.73**	**0.69**	**0.64 to 0.73**	Same
**Birth interval**	First birth as baseline								
Within 24 months	−**2.07**	−**2.45 to 1.68**	**0.35**	**0.27 to 0.45**	***0.37***	***0.28* to *0.47***	***0.26***	***0.21* to *0.34***	Same
2–3 years	−**0.74**	−**1.20 to 0.27**	0.79	0.60 to 1.04	***0.71***	***0.54* to *0.94***	***0.44***	***0.34* to *0.56***	Different
More than 3 years	0.33	−0.11 to 0.76	**1.72**	**1.29 to 2.30**	*1.17*	*0.88* to *1.56*	***0.71***	***0.55* to *0.91***	Different
**Gestation, months**	−0.06	−0.15 to 0.03	0.97	0.92 to 1.02	**0.96**	**0.92 to 1.00**	**0.96**	**0.92 to 1.00**	Different
**Socioeconomic status**	Poorest 20% as baseline								
Second-poorest 20%	−0.13	−0.58 to 0.31	0.86	0.68 to 1.08	0.94	0.77 to 1.15	0.94	0.77 to 1.15	Same
Middle 20%	−0.13	−0.53 to 0.26	0.87	0.69 to 1.10	*1.08*	*0.86* to *1.35*	*0.89*	*0.72* to *1.11*	
Next-richest 20%	0.31	−0.16 to 0.79	1.07	0.84 to 1.36	1.22	0.99 to 1.49	1.22	0.99 to 1.49	
Richest 20%	0.33	−0.23 to 0.89	1.01	0.78 to 1.31	1.22	0.98 to 1.52	1.22	0.98 to 1.52	
**Previous depression**	None as baseline								
1 or 2, < 2 weeks	−**0.86**	−**1.27 to 0.44**	**0.55**	**0.44 to 0.67**	**0.63**	**0.53 to 0.75**	**0.63**	**0.53 to 0.75**	Same
One, ≥ 2 weeks	−**1.35**	−**1.95 to 0.74**	**0.38**	**0.3 to 0.48**	**0.52**	**0.43 to 0.63**	**0.52**	**0.43 to 0.63**	
Both, ≥ 2 weeks	−**1.65**	−**2.55 to 0.75**	**0.34**	**0.17 to 0.68**	0.59	0.34 to 1.03	0.59	0.34 to 1.03	Different
**Distance to health facility**	<2.5 km as baseline								
2.5–4.99 km	0.15	−0.37 to 0.67	**1.38**	**1.03 to 1.86**	1.04	0.84 to 1.30	1.04	0.84 to 1.30	Different
5–7.49 km	0.22	−0.49 to 0.93	**1.37**	**1.02 to 1.86**	1.100	0.90 to 1.36	1.10	0.90 to 1.36	
More than 7.5 km	0.18	−0.40 to 0.76	1.26	0.91 to 1.74	1.09	0.88 to 1.36	1.09	0.88 to 1.36	Same
**Religion**	Non-Catholic Christian as baseline								
Catholic	−0.11	−0.44 to 0.21	0.96	0.81 to 1.13	0.93	0.81 to 1.06	0.93	0.81 to 1.06	Same
Muslim	−0.05	−0.87 to 0.77	0.77	0.38 to 1.54	0.86	0.48 to 1.57	0.86	0.48 to 1.57	
Other	0.09	−0.38 to 0.56	1.02	0.53 to 1.93	***2.04***	***1.04* to *3.99***	*0.81*	*0.46* to *1.44*	Different
**Tribe**	Chewa as baseline								
Ngoni	−0.56	−1.20 to 0.07	**0.7**	**0.51 to 0.95**	0.76	0.59 to 0.98	0.76	0.59 to 0.98	Different
Senga	**1.16**	**0.50 to 1.83**	0.97	0.57 to 1.64	**1.97**	**1.44 to 2.69**	1.97	1.44 to 2.69	Different
Yao	0.17	−0.56 to 0.90	1.17	0.58 to 2.33	1.18	0.65 to 2.14	1.18	0.65 to 2.14	Same
Other	0.10	−1.05 to 1.24	1.12	0.61 to 2.07	1.03	0.62 to 1.72	1.03	0.62 to 1.72	
ρ	ρ=0 not rejected		0.08	0.04 to 0.14	Panel variables not possible				

**Note:** Figures in bold denote significance (*P*<0.05); figures in italics show the variables for which the proportional odds assumption had to be relaxed, meaning they have different ORs across the two cut points.

**Abbreviations:** OR, odds ratio; CI, confidence interval.

**Table 4:** Findings from the univariate analyses of the original UK data for the four regression models

Variables	Linear		Log plan		LMUP all		LMUP 3		Model comparison
	β-Coefficient	95% CI	OR	95% CI	OR	95% CI	OR	95% CI	
**Mother’s age, years**	30–39 as baseline								
<20	−**5.40**	−**6.19 to** −**4.61**	**0.05**	**0.03 to 0.1**	**0.10**	**0.07 to 0.15**	**0.08**	**0.06 to 0.13**	Same
20–29	−**2.63**	−**3.13 to** −**2.14**	**0.29**	**0.22 to 0.38**	**0.31**	**0.24 to 0.39**	**0.27**	**0.21 to 0.35**	
≥40	−**1.34**	−**2.52 to** −**0.17**	0.56	0.30 to 1.07	0.62	0.34 to 1.12	**0.54**	**0.29 to 1.00**	Different
**Child order**	First child as baseline								
Second	**1.83**	**1.36 to 2.46**	**1.87**	**1.40 to 2.49**	**1.66**	**1.30 to 2.13**	**1.99**	**1.51 to 2.63**	Same
Third or more	0.84	0.61 to 1.17	0.73	0.52 to 1.01	0.76	0.58 to 1.01	0.85	0.63 to 1.14	
**Education level**	School as baseline								
Post-16 years old	0.14	−0.54 to 0.82	1.03	0.74 to 1.45	1.07	0.80 to 1.42	1.05	0.77 to 1.42	Same
Higher	**1.43**	**0.77 to 2.08**	**2.12**	**1.53 to 2.93**	**1.9**	**1.43 to 2.52**	**1.94**	**1.43 to 2.62**	
**Living with**	Living with husband as baseline								
Partner	−**2.99**	−**3.47 to** −**2.52**	**0.21**	**0.16 to 0.30**	**0.21**	**0.16 to 0.28**	**0.20**	**0.14 to 0.27**	Same
Not husband/partner	−**6.64**	−**7.11 to** −**6.17**	**0.02**	**0.01 to 0.03**	**0.03**	**0.02 to 0.05**	**0.03**	**0.02 to 0.05**	
**Ethnicity**	White British as baseline								
White other	−0.41	−1.19 to 0.38	0.83	0.56 to 1.21	0.78	0.55 to 1.09	0.75	0.52 to 1.09	
Asian	0.82	−0.21 to 1.86	1.58	0.93 to 2.69	0.98	0.66 to 1.48	1.64	0.98 to 2.74	Same
Black	−**2.23**	−**3.13 to** −**1.34**	**0.32**	**0.19 to 0.51**	**0.40**	**0.28 to 0.58**	**0.41**	**0.27 to 0.60**	
Mixed/other	−0.85	−2.12 to 0.42	**0.48**	**0.25 to 0.92**	0.62	0.37 to 1.04	**0.56**	**0.32 to 0.98**	Different

**Note:** Figures in bold denote significance (*P*<0.05).

**Abbreviations:** LMUP, London Measure of Unplanned Pregnancy; OR, odds ratio; CI, confidence interval.

**Table 5:** Comparison of four multivariate regression models using the original UK data

Variables	Linear		Log plan		LMUP all		LMUP 3		Model comparison
	β-Coefficient	95% CI	OR	95% CI	OR	95% CI	OR	95% CI	
**Mother’s age, years**	30–39 as baseline								
<20	−**2.16**	−**2.95 to** −**1.37**	**0.19**	**0.08 to 0.44**	**0.34**	**0.21 to 0.53**	**0.26**	**0.15 to 0.45**	Same
20–29	−**1.25**	−**1.70 to** −**0.80**	**0.40**	**0.27 to 0.58**	**0.53**	**0.40 to 0.70**	**0.40**	**0.29 to 0.55**	
≥40	−0.43	−1.40 to 0.54	0.91	0.40 to 2.07	0.95	0.51 to 1.77	0.81	0.40 to 1.67	
**Child order**	First child as baseline								
Second	−0.01	−0.46 to 0.43	1.11	0.75 to 1.65	0.98	0.75 to 1.29	1.20	0.85 to 1.68	Same
Third or more	−**2.19**	−**2.74 to** −**1.65**	**0.21**	**0.13 to 0.33**	**0.28**	**0.20 to 0.40**	**0.26**	**0.17 to 0.38**	
**Education level**	School as baseline								
Post-16 years old	−0.25	−0.76 to 0.25	0.80	0.51 to 1.24	0.90	0.66 to 1.22	0.84	0.58 to 1.21	Same
Higher	−0.46	−0.99 to 0.06	0.87	0.56 to 1.37	0.81	0.59 to 1.11	0.72	0.49 to 1.06	
**Living with**	Living with husband as baseline								
Partner	**3.21**	**2.64 to 3.78**	**11.32**	**5.57 to 23.00**	**5.43**	**3.79 to 7.79**	**4.94**	**3.34 to 7.32**	Same
Not husband/partner	**6.06**	**5.51 to 6.61**	**51.85**	**25.59 to 105.05**	**26.66**	**18.22 to 39.01**	**25.61**	**16.82 to 38.99**	
**Ethnicity**	White British as baseline								
White other	−0.22	−0.82 to 0.38	0.87	0.52 to 1.48	0.79	0.55 to 1.12	0.73	0.48 to 1.13	Same
Asian	−0.37	−1.18 to 0.44	0.99	0.52 to 1.91	**0.57**	**0.36 to 0.90**	0.85	0.47 to 1.55	Different
Black	0.01	−0.68 to 0.69	0.81	0.41 to 1.60	1.10	0.73 to 1.67	1.05	0.65 to 1.71	Same
Mixed/other	−0.79	−1.73 to 0.15	**0.31**	**0.14 to 0.69**	**0.55**	**0.32 to 0.94**	**0.42**	**0.22 to 0.80**	Different

**Note:** Figures in bold denote significance (*P*<0.05).

**Abbreviations:** LMUP, London Measure of Unplanned Pregnancy; OR, odds ratio; CI, confidence interval.
